# Generation of affibody molecules specific for HPV16 E7 recognition

**DOI:** 10.18632/oncotarget.12174

**Published:** 2016-09-21

**Authors:** Xiangyang Xue, Bingbing Wang, Wangqi Du, Chanqiong Zhang, Yiling Song, Yiqi Cai, Danwei Cen, Ledan Wang, Yirong Xiong, Pengfei Jiang, Shanli Zhu, Kong-Nan Zhao, Lifang Zhang

**Affiliations:** ^1^ Department of Microbiology and Immunology, Institute of molecular virology and immunology, Wenzhou Medical University, Wenzhou, China; ^2^ Department of General Surgery, First Affiliated Hospital, Wenzhou Medical University, Wenzhou, China; ^3^ Department of Obstetrics and Gynecology, Second Affiliated Hospital, Wenzhou Medical University, Wenzhou, China

**Keywords:** cervical cancer, human papillomavirus, E7, affibody molecules, in vivo imaging

## Abstract

Cervical cancer caused by infection with high-risk human papillomavirus remains to be the most deadly gynecologic malignancy worldwide. It is well documented that persistent expression of two oncogenes (E6/E7) plays the key roles in cervical cancer. Thus, *in vivo* detection of the oncoproteins is very important for the diagnosis of the cancer. Recently, affibody molecules have been demonstrated to be a powerful targeting probe for tumor–targeted imaging and diagnosis. In this study, four HPV16 E7-binding affibody molecules (Z_HPV16 E7_127, Z_HPV16E7_301, Z_HPV16E7_384 and Z_HPV16E7_745) were screened from a phage-displayed peptide library and used for molecular imaging in tumor-bearing mice. Biosensor binding analyses showed first that the four affibody molecules bound to HPV16 E7 with very high affinity and specificity. They co-localized with E7 protein only in two HPV16-positive cancer cells (SiHa and CaSki). Furthermore, affibody Z_HPV16E7_384 was conjugated with Dylight755 and used for *in vivo* tumor-imaging. Strongly high-contrast tumor retention of this affibody only occurred in HPV16-derived tumors of mice as early as 30 min post-injection, not in HPV-negative and HPV18-derived tumors. The accumulation of Dylight755-conjugated Z_HPV16E7_384 in tumor was achieved over a longer time period (24 h). The data here provide strong evidence that E7-specific affibody molecules have great potential used for molecular imaging and diagnosis of HPV-induced cancers.

## INTRODUCTION

Cervix carcinoma (CxCa) caused by infection with high-risk human papillomavirus (HR-HPV) remains to be the most deadly gynecologic malignancy worldwide despite global efforts to prevent this disease by early screening, diagnosis and treatment in the past decades [1]. An accurate diagnosis of cervical cancer, especially the specific detection of tumor metastasis and invasion, is essential for determining treatment of cancer patients and predicting the clinical outcome. Persistent infection of HR-HPVs including HPV 16, 18, 31, 33, 35, 39, 45, 51, 52, 56, 58, and 59 has been demonstrated to be the major etiological cause of CxCa [2, 3], with that HPV16 infection alone contributes to over 50% cancer cases [4]. Thus, HPV-based screening is very important for predicting the incidence of invasive cancer. Recently, Ronco *et al*. reported that HPV-based screening could provide 60–70% greater prediction of invasive CxCa compared with cytology-based screening [5]. Standard surgical treatment of cervical cancer consists of radical hysterectomy combined with bilateral pelvic lymphadenectomy. However, no method can be used for the specifically intraoperative detection for invasive cancer with metastasis status, parametrial involvement, lymphovascular space invasion and deep cervical stromal invasion.

It has been demonstrated that HPV genomes integrate into the host chromosome, leading to viral E2 gene disruption and persistent expression of E6/E7 oncogenes, which are the key events in cervical carcinogenesis [6–8]. E6 oncoprotein blocks the function of tumor suppressor p53, whereas E7 leads to cellular transformation by targeting pRb, thus contributing to cervical carcinogenesis [9–12]. E6/E7 oncoproteins also target multiple signal molecules by regulating different signaling pathways that are equally important for transformation [13–17]. Therefore, E6/E7 are the ideal molecular targets for diagnostic strategies against HPV-associated neoplasia due to they are specifically expressed in HPV infected tissues.

Affibody molecules are a novel class of small single-domain proteins (6.5 kDa) based on non-immunoglobulin scaffolds of the three-helix bundle motif of the Z domain derived from staphylococcal protein A, which can be isolated for high affinity and specificity to any given protein target [18]. The affibody molecules provide rapid tumor localization and fast clearance from nonspecific compartments. Currently, affibody molecules are the very attractive substitutes for full-size antibodies in biotechnological applications, *in vivo* imaging and cancer targeted therapy due to their small size and low immunogenicity [18]. Several high affinity affibody molecules targeting many tumor-associated proteins have been generated over the last few years. These proteins include human epidermal growth factor receptor 2 (HER2) [19], epidermal growth factor receptor (EGFR) [20] and insulin-like growth factor type 1 (IGF1R) [21].

In this report, we describe screening and characterization of four HPV16 E7-binding affibody molecules and their application to *in vivo* molecular imaging in tumor-bearing mice. Four potential affibody molecules (Z_HPV16 E7_127, Z_HPV16E7_301, Z_HPV16E7_384 and Z_HPV16E7_745) were screened from phage display library by panning, ELISA screening and DNA sequencing. After confirming the affinity and specificity of these selected affibody molecules in binding to HPV16 E7, affibody Z_HPV16E7_384 was conjugated with Dylight755 dyes. This Dylight755-conjugated affibody was further accessed for the application to *in vivo* image HPV16-positive tumor in mice. To our knowledge, this is the first time to report that HPV16 E7-specific affibody is a novel probe used for *in vivo* imaging and diagnosis of HPV16-positive tumor.

## RESULTS

### Selection of HPV16 E7-binding affibody molecules

**Figure 1:**
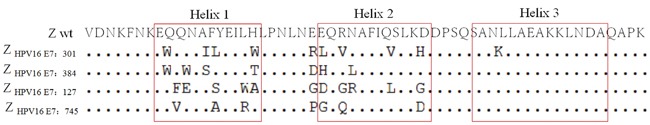
Amino acid sequence alignment of wild-type Z domain and four selected affibody molecules Three α-helices in the wild-type Z domain are boxed and randomized amino acid residues are presented. Horizontal dots indicate amino acid identities.

**Figure 2:**
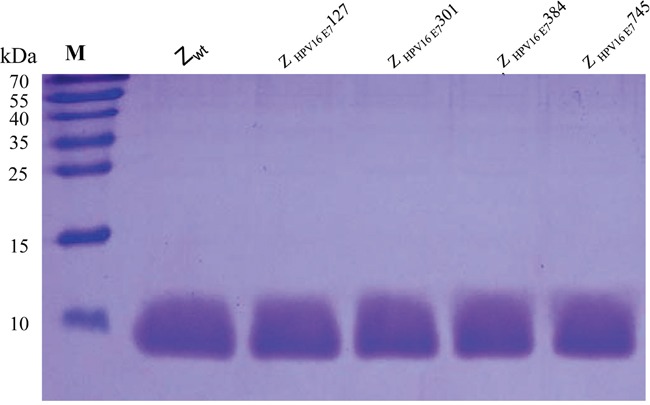
SDS-PAGE of 4 purified HPV16 E7-binding affibodies Protein bands were visualized with Coomassie Brilliant Blue staining. Lane 1, Z wt; lane 2, Z_HPV16E7_127; lane 3, Z_HPV16E7_301; lane 4, Z_HPV16E7_384 and lane 5, Z_HPV16E7_745; M, marker molecular masses.

One hundred fifty clones that showed significantly higher interaction with HPV16 E7 were selected for DNA sequencing after four-round panning of bacteriophage display and following an ELISA screening for target-binding activity (Supplementary Figure S1). Four potential HPV16 E7-binding affibody molecules: Z_HPV16E7_127, Z_HPV16E7_301, Z_HPV16E7_384 and Z_HPV16E7_745, which showed the highest ranking of binding affinity in the ELISA screening were selected for sequence homologous analysis. Results showed that the four molecules had a high homology in framework region of the affibody, but were highly diverse in the helical regions (Figure 1). Several clones with high binding affinity, such as clone 921, 992, 1037, et al. were discarded because there were one or two mutations in framework region of the affibody. The four affibody genes were subsequently inserted into a pET21a (+) vector to construct four affibody gene expression plasmids. The four affibody molecules expressed in *E. coli* were purified by Ni-NTA agarose affinity chromatography. The purity of the final products was approximately 95% for these recombinant proteins determined by SDS-PAGE with Coomassie blue staining (Figure 2).

### Biosensor binding analyses of the selected affibody molecules

**Figure 3:**
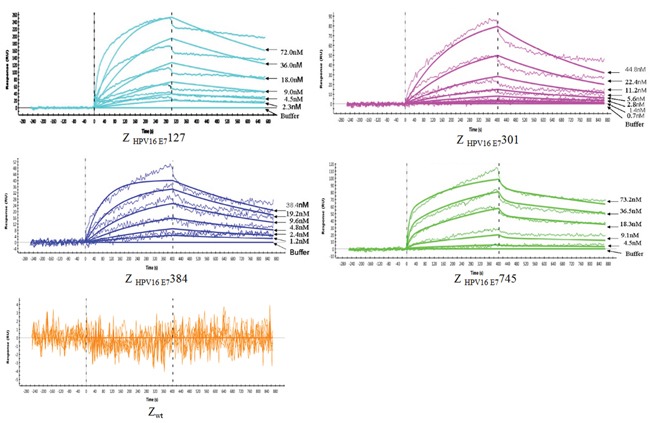
Biosensor binding analysis of 4 purified affibody molecules Sensorgrams obtained after injection of the Z_HPV16E7_127, Z_HPV16E7_301, Z_HPV16E7_384 and Z_HPV16E7_745 affibody molecules with different concentrations over a sensor chip flow-cell surface containing HPV16 E7 protein. The wild Z wt molecule was set as control. All samples were run in duplicates, and the response obtained from an activated and deactivated reference surface has been subtracted from all curves.

Affinity is the most important property for tumor-targeting imaging agent. Thus, the affinities of 4 affibody molecules and wild type SPA-Z scaffold (Z wt) affibody molecule based on their binding to recombinant HPV16 E7 were firstly analyzed (Figure 3). All 4 affibody molecules have good binding profiles. The dissociation equilibrium constants (KD) of Z_HPV16E7_127, Z_HPV16E7_301, Z_HPV16E7_384 and Z_HPV16E7_745 were 4.82×10^−5^, 2.21×10^−6^ mol/L, 2.20×10^−6^ mol/L and 1.80×10^−6^ mol/L mol/L, respectively, which were significantly lower than that of Zwt affibody (3.75×10^−2^ mol/L). In contrast, the association rate constants (ka) of the 4 affibody molecules were significantly higher than that of Z wt affibody.

### Analysis of affibody interaction with native HPV16 E7 protein

**Figure 4:**
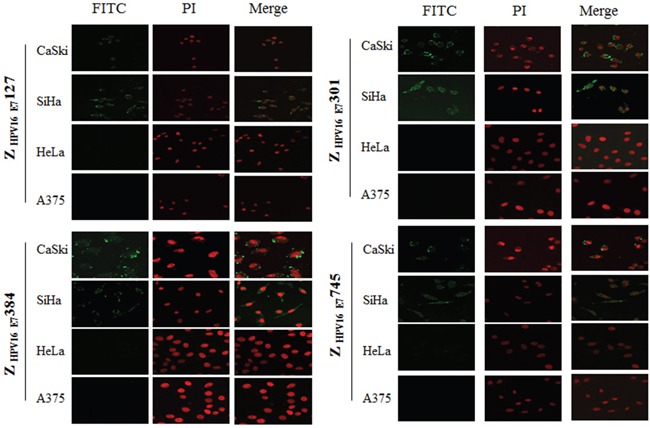
Fluorescence staining of HPV16 positive SiHa and CaSki cells with Z_HPV16E7_127, Z_HPV16E7_301, Z_HPV16E7_384 and Z_HPV16E7_745 affibody molecules Combined with FITC-conjugated goat anti-mouse IgG, His-tag McAb which recognized His-tag of affibody molecules was used to detect the affibody molecules. The brightly dotted or crumby fluorescence signals were observed in both perinuclear area and nuclear membrane (200×). HPV-negative A375 cell and HPV18 positive HeLa cell were used as controls and did not show any fluorescence signal when the cells were stained with the 4 selected HPV16 E7-binding affibodies. Nuclei were counterstained with PI staining (red).

We next determined whether the 4 HPV16 E7-binding affibody molecules could also bind to the native HPV16 E7 protein. HPV16 positive SiHa and CaSki cells labeled with the 4 HPV16 E7-binding affibody molecules showed brightly dotted or crumby fluorescence signals in both perinuclear area and nuclear membrane, similar to the pattern of anti-HPV16 E7 polyclonal antibody labelling while no signal could be observed in cells labelled with Z wt antibody molecule (Figure 4, Supplementary Figure S2, Supplementary Figure S3, and Supplementary Figure S4). Few differences were observed among the four selected affibody molecules. Furthermore, both HPV18 positive HeLa cells and HPV negative A375 cells did not show any fluorescence signal when the cells were stained with the 4 selected HPV16 E7-binding affibodies (Figure 4 and Supplementary Figure S2). These data further confirmed that the 4 affibody molecules could specifically bind to HPV16 E7 and did not cross-react with other HPV types.

### Biodistribution and tumor-targeted fluorescence imaging of Z_HPV16E7_384 affibody in tumor-bearing mice *in vivo*

**Figure 5:**
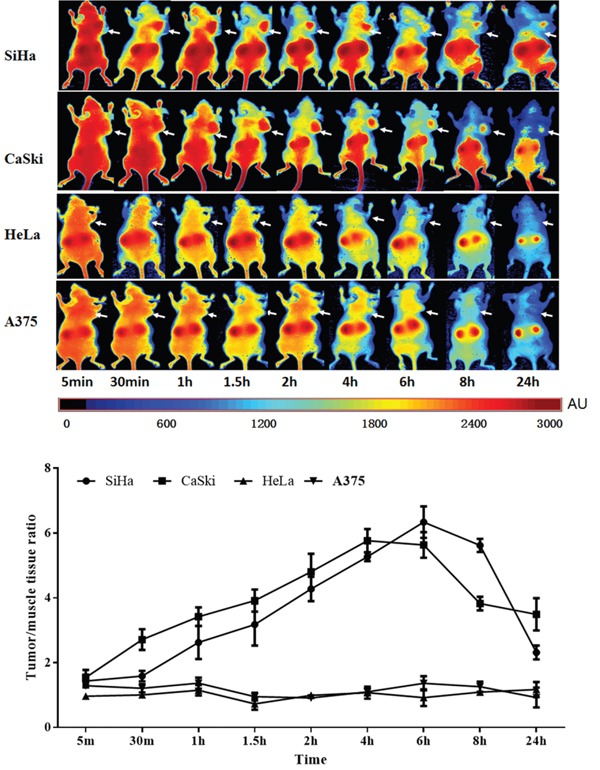
Tumor-targeted fluorescence imaging of the Z_HPV16E7_384 affibody **A.** *In vivo* fluorescence imaging of tumor-bearing mice (arrows) injected with Dylight755-conjugated Z_HPV16E7_384 affibody at 5 min, 0.5, 1, 1.5, 2, 4, 6, 8 and 24 h. **B.** Tumor-to-background ratio of mice injected with Dylight755-conjugated Z_HPV16E7_384 affibody. The data was represented as mean ± standard deviation (SD) of three mice (n = 3) in each group.

We then focused on investigating the *in vivo* imaging property of Z_HPV16E7_384 affibody conjugated with Dylight755 in animal experiments. Dylight755-conjugated Z_HPV16E7_384 (10 μg in 50 μl per mouse) was intravenously injected into the athymic nude mice bearing HPV16-, HPV 18- and HPV-negative-derived subcutaneous tumors. An *in vivo* fluorescence imaging system based on near-infrared fluorescence signal was used to determine *in vivo* biodistribution and tumor-retention efficacy of the Z_HPV16 E7_384 affibody over a time course of 5 min to 24 h. The fluorescent signals were obtained from the wavelength of 730 nm to 950 nm at 10 nm interval. We observed that fluorescence signal derived from the Dylight755-conjugated Z_HPV16 E7_384 occurred in tumor position of HPV16-positive mice (injected with either SiHa or CaSki cells) as early as 30 min post-injection (p.i.) (Figure 5). The strength of fluorescence signal reached to a peak in tumor of the mice derived from CaSki injection at 4 h p.i. and from SiHa injection at 6 h p.i., respectively. After that, the fluorescence signal decreased in both CaSki- and SiHa-derived tumors over the 24-h time course (Figure 5).

Tumor specific retention of the Dylight755-conjogated Z_HPV16E7_384 affibody was still obvious in HPV16 positive mice at 24 h p.i. Non-specific accumulation in the kidneys was noticed due to proteins with size below the renal filtration threshold (Supplementary Figure S5). No fluorescence signal in the tumor position was observed in mice injected with Dylight755-labeled wild SPA-Z affibody molecules, neither in A375 tumor-bearing mice and nor HPV18 positive tumor-bearing mice (HeLa cell injection) (Figure 5 and Supplementary Figure S6). All the data provide evidence that the affinity of Z_HPV16E7_384 affibody was specific to the HPV16 E7-derived tumor.

**Figure 6:**
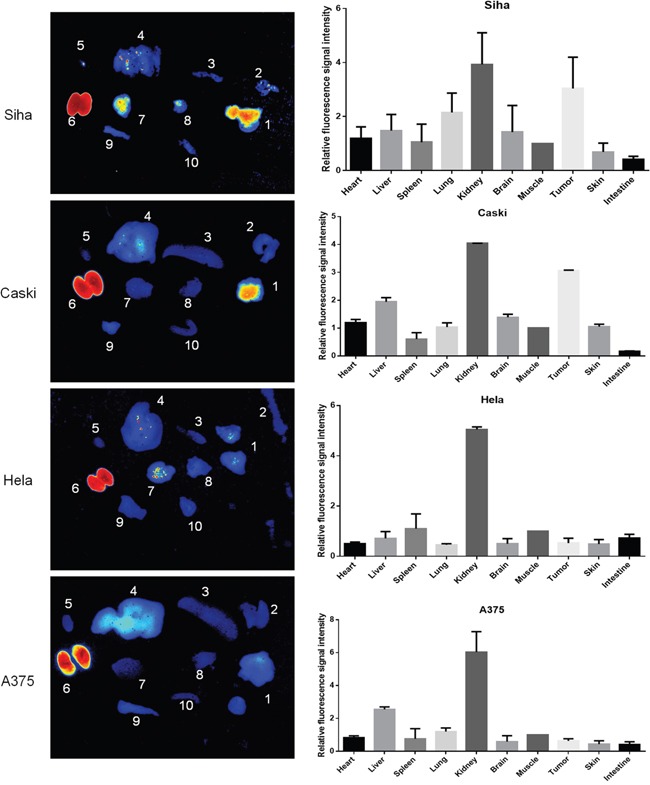
*Ex vivo* imaging of the fluorescence retention of Dylight755-labelled Z_HPV16E7_384 affibody in different tissues **A.** Fluorescence imaging of tumor *ex vivo* at 24 h p.i. of Dylight755- conjugated Z_HPV16E7_384 affibody injection. In each image: 1, tumor; 2, skin; 3, spleen; liver; 5, heart; 6, kidney; 7, brain; 8, lung; 9, muscle; 10, intestine. **B.** The relative fluorescence signal intensity of *ex vivo* imaging in the tumors and major organs at 24 h p.i. of Dylight755-labelling Z_HPV16 E7_384 affibody injection. The value= fluorescence signal intensity in the tumors and other major organs / fluorescence signal intensity in the corresponding muscle tissue.

To confirm that the *in vivo* fluorescence imaging retention is only present in tumor position, mice were sacrificed for investigating the fluorescence signal intensity in *ex vivo* tumors and nine major organs at 24 h p.i. *Ex vivo* imaging confirmed that the strong fluorescence signal only occurred in tumors derived from the injection of HPV16 positive cancer cells (Figure 6). No fluorescence signal was detected in both HPV18 positive and HPV16 negative mice. Relative fluorescent intensity (RFI) [22] is highly retained in HPV16-derived tumors. For example, the RFIs in SiHa and CaSki-derived tumors were 3.04±1.16 and 3.05±0.03, which are significantly higher than those (0.64±0.13% and 0.53±0.19) in HeLa and A375-bearing tumors. These data further provide evidence that the selected HPV16 E7-binding affibody was highly specific to the retention of fluorescence signal in tumor position.

## DISCUSSION

Since the first HER2 binding affibody molecule discovered [23, 24], a serial of affibody molecules targeting different proteins including EGFR, IGF-1R and HIV-1-gp120 have been reported [20, 25, 26]. The affibody molecules are small size proteins. Their size is less than one of third of scFv if without cysteine residues. Thus, they can be produced by conventional peptide synthesis methods. In this study, we have successfully screened four small affibody molecules from phage displayed peptide library and produced them in an *E. coli* BL21 expression system. These small size affibody molecules are suitable for addressing multiple protein targets by providing rapid tumor localization and fast clearance from nonspecific compartments. The affibody molecules are highly soluble and stable. The Z-domain scaffold in the affibody molecules provides structural rigidity and conformational stability, which are crucial for efficient binding to target [27]. Preclinical studies have demonstrated the potential of affibody molecules for specific and high-contrast radionuclide imaging of HER2 *in vivo*. Pilot clinical data using (18)F, indium-111 and gallium-68 labeled anti-HER2 affibody tracer have confirmed its utility for radionuclide imaging in cancer patients [24, 27]. Thus, the four potential affibody molecules produced in this study with high stability have the potential utility in molecular imaging, which have been highlighted in animal study.

Molecular imaging shows promise as a useful tool to aid drug discovery and development and also to provide important prognostic and predictive diagnostic information affecting patient management in the clinic. However, the use of molecular imaging used for diagnostics has not been widely adopted in CxCa, in part due to the lack of suitable targeting agents that have higher target-binding affinity and specificity. Target-binding affinity is an important feature of a molecular agent for its successful tumor-targeting *in vivo*. The agent should highly bind to the targeted molecules associated with tumor tissues and with minimal binding to normal tissues. Both polyclonal and monoclonal antibodies are traditionally used as affinity reagents in diagnostic and biological applications. As a new class of affinity ligands, affibody molecules selected from combinatorial libraries have higher affinity, based on the 58-amino acid, cysteine-free, three-helix bundle Z-domain as scaffold [28, 29]. Here, we demonstrated that the four affibody molecules bind to HPV16 E7 protein with very high affinity. HR-HPV E7 protein as an oncoprotein interacts not only with the proteins of pRb family but also with many other proteins involved in multiple signaling and immune response pathways [15–17]. E7 also prevents G1 arrest in response to a variety of anti-proliferative signals to play a major role in cervical neoplasia. Blocking expression of HR-HPV E7 and disruption of its downstream pathways have previously been proven a successful approach for inhibition of tumor cell growth in cervical cancers [30–35]. However, this approach requires the binders to HPV E7 with stable and high-affinity for successful imaging of HPV-associated cancers. Therefore, the identified four affibody molecules have great advantages in predictive and prognostic diagnostics of cervical neoplasia.

Highly specificity of a targeting agent used for imaging is the other important property, which makes a high contrast in tumor within a few hours after injection [27]. In the present study, biodistribution analysis showed that the selected Z_HPV16E7_384 affibody could not only co-localize with E7 oncoprotein in HPV16-positive cancer cells *in vitro*, but also quickly and specifically accumulate in the tumor position of HPV16-bearing animals *in vivo*. Orlova and colleagues have reported that one HER2-specific affibody molecule Z_HER2_342 had a better uptake into tumor and provided higher tumor-to-blood ratios than HER2-specific scFv antibody fragment in the imaging of HER2-positive SKOV-3 xenografts [19]. Similar to the previous reports [19, 36], our study also found that Z_HPV16E7_384 affibody conjugated with dylight755 showed highly instant uptake into tumor tissues when used for *in vivo* imaging in mice model. Although the kidney of treated animals also had a higher uptake of Z_HPV16 E7_384 affibody, renal failure detection did not show any obvious cytotoxic effects occurred in kidneys of the animals (data not shown). Currently, the prognosis in advanced-stage cervical cancer remains poor. Nonetheless, the four affibody molecules generated in the present study used for tumor-specific intraoperative fluorescence imaging may improve staging and debulking efforts in cytoreductive surgery and thereby improve prognosis.

In conclusion, for the first time, we have identified and produced four affibody molecules which bind to HPV16 E7- protein with very high affinities and specificity. The four potential affibody molecules co-localized with E7 protein in HPV16-positive cancer cells and only accumulated in the tumor position of HPV16-bearing animals. Therefore, the affibody molecules identified and produced in this study may have great potential for molecular imaging in cervical cancer caused by HPV16 infection.

## MATERIALS AND METHODS

### Construction of a phage display library containing staphylococcal protein A (SPA) derived-Z domain scaffold

The random affibody library was created by PCR amplification from a wild SPA-Z scaffold template by using the random primers encoding helices 1 and 2 of the Z domain. The gene fragments were then digested with Sfi I and Not I restriction endonuclease and cloned into pCANTAB5E phagemid vector to construct recombinant pCANTAB5E/SPA-N vector. The recombinant vectors were then transformed into *E. coli* TG1 cells. The naive library of affibody molecules cloned into vector was about a complexity of 1*10^9^ and with 100% diversity in SPA-Z scaffold. After evaluated the randomness and capacity of inserted affibody library, the phage stocks then resuspensed in PBS/glycerol solution to a final approximate concentration of 20% glycerol, aliquoted and stored at −80°C. Phage particles (nondisplaying) for the infection of target cells were prepared according to standard procedures using helper phage M13K07, and each selection was done accordingly.

### Selection of potential affibody molecules binding to HPV16 E7 with high affinity

In our previous study, a recombinant HPV16 E7 protein with high purity has been prepared and was used as panning target protein during selections [37]. Phage selection of binders to HPV16 E7 was performed in the well of ELISA plate. Firstly, the target protein of 10ug/ml (200ul/ well) in carbonate coating buffer was coated into ELISA plates (Milierepore) overnight at 4°C. The unbound HPV16 E7 protein in ELISA plates was washed. After block with 3% nonfat milk for 1 hour, these wells could be used for the further panning. Secondly, the library was subjected to four rounds of selection in solution using a 2-fold decreasing target concentration for each round. The phage library was subjected to biopanning against HPV16 E7 for 1 hour and 45 minutes at room temperature under continuous rotation. For each round of selection, the wells were washed three times with 5% nonfat milk in PBS supplemented with 0.1% Tween 20 (PBST) at room temperature under continuous rotation for 30 min. Thirdly, ELISA-based ranking was used to further test their affinities to target protein. The supernatants containing potential affibody molecules were loaded in microtiter wells, which had been previously coated with 10 μg/mL HPV16 E7 protein and blocked with 3% non-fat milk powder in PBST for 1 hour at room temperature. The plates were washed four times with PBST prior to the addition of 100 μL of 1:10000 diluted rabbit anti-M13 polyclonal antibodies per well and incubated for 1 hour. After washing the wells four times, 100 μL horseradish peroxidase (HRP) -conjugated goat anti-rabbit IgG (1:5,000) per well were added and incubated for 1 hour. The wells were washed four times and 100 μL developing TMB solution was added to each well. After 30 minutes, 100 μL of the stop solution (2 M H2SO4) was added to each well. The absorbance (OD) was measured at 450 nm by using a Bio-tek ELISA microplate reader. The phages with the higher signal of OD450 absorbance value were selected. The sequences of inserted fragments in selected phage were act as potential affibody molecules with high affinity, which specifically binding HPV16 E7 protein.

### Expression and purification of HPV16 E7-binding affibody molecules

The sequences of selected HPV16 E7-binding affibodies molecules, including Z_HPV16 E7_127, Z_HPV16 E7_301, Z_HPV16 E7_384, Z_HPV16 E7_745 and wild SPA-Z scaffold (Z_-WT_), were subcloned into the *Nde I* and *EcoRI* sites of pET21a(+) expression vector. Following confirmation of the inserted sequences by enzyme digestion and DNA sequencing, positive plasmids were transformed into *E. coli* BL21 (DE3) for expression of the fusion proteins. The 6×His-tagged recombinant proteins were analyzed by sodium dodecyl sulfate-polyacrylamide gel electrophoresis (SDS-PAGE) and confirmed by Western blotting using anti-His antibody (Sigma). The recombinant protein was purified by affinity chromatography using precharged Ni-NTA Sepharose column (Qiagen) and refolded when dialyzed in PBS using Slide-A-Lyzer (Pierce) according to the manufacturer's recommendations. The purity of purified proteins was verified by SDS-PAGE, and the protein concentrations were determined by the bicinchoninic acid (BCA) protein quantitation method.

### Biosensor analyses of the interaction between affibody molecules and HPV16 E7 protein

A ProteOn XPR36 instrument (Bio-Rad) was used for real-time biospecific interaction analysis between selected affibody molecules and the target protein. HPV16 E7 protein diluted in 10 mmol/L NaAc (pH 4.5) was immobilized into the surface of carboxylate glucans in HTG sensor chip according to the manufacturer's instructions. Another flow-cell surface was activated and deactivated to be used as a reference controls. Different concentration of Z_HPV16 E7_127, Z_HPV16 E7_301, Z_HPV16 E7_384 and Z_HPV16 E7_745, ranging from 1.0 nM to 64.0 nM, were injected over all surfaces with a flow rate of 30 μL/min. Wild SPA-Z (Zwt) affibody was set as negative control. Binding analyses were done at 25°C, and PBS was used as the running buffer. The dissociation equilibrium constant (KD), the association rate constant (ka), and the dissociation rate constant (kd) were calculated using BIA evaluation 3.0.2 software (Biacore). A one-to-one Langmuir binding model was used to assume the mass transfer effects into account.

### Immunofluorescence detection

Human cervical cancer cells, including HPV16 positive SiHa cell (ATCC: HTB-35) and CaSki cell (ATCC: CRL-1550), HPV18 positive HeLa (ATCC: CCL-2) and HPV negative melanoma cell of A375 (ATCC: CRL-1619) were cultured on multi-well slides at 37°C. After gently removed the medium, cells were stained for 6 hour with the HPV16 E7-binding affibody molecules or wild SPA-Z (Zwt) control with final concentration of 50μg/ml. After staining, cells were fixed with 4% paraformaldehyde at room temperature for 10 min and permeabilized by 0.3% Triton X-100 at room temperature for 10 min. After blocked in RPMI-1640 containing 10 % FBS for 60 min at 37°C, the cells were used the detection of HPV16 E7-binding affibody molecules with mouse anti-His monoclonal antibody, rabbit anti-affibody (wild SPA-Z) serum (prepared in-house), followed the addition of secondary antibodies FITC-conjugated goat anti-mouse and goat anti-rabbit IgG (H+L) (Invitrogen) at room temperature for 1h. Cell nucleuses were stained with 50μg/mL propidium iodide (PI) (MultiSciences Biotech Co,.Ltd China) at room temperature for 5min. The images were visualized in a confocal fluorescence microscope (Nikon C1-i, Japan).

To observe the location of HPV16 E7, rabbit anti-HPV16 E7 antibodies (prepared in-house) [37] and FITC-conjugated goat anti-rabbit IgG (H+L) antibody (Invitrogen) were used to stain cells. The images were visualized in a confocal fluorescence microscope (Nikon C1-i, Japan).

### Labeling of affibody molecules with Dylight755

The labeling with Dylight755 of the HPV16 E7-binding affibody molecules was done according to the manufacture's instruction. The labeled affibody molecules were dialyzed in cold PBS containing 2% dicarbonate, 1mmol/L EDTA.2Na to remove the surplus of Dylight755 dye and further analyzed using Biacore technology to verify that the labeling procedure had not affected the binding affinity to HPV16 E7. The labelling efficiency was detected in the wavelength of 730-950nm by *in vivo* Fluorescence Imaging System (CRi Maesro 2.10). The labeled affibody molecules then resuspensed in PBS to a final approximate concentration of 1ug/ul and stored at −20°C for further use.

### Biodistribution of HPV16 E7-binding affibodies in tumor-bearing mice

Female nude mice (6-7 week old, BALB/c), purchased from the shanghai Slac laboratory animal CO. LTD (Shanghai, China), were used to establish the SiHa, CaSki, HeLa cervical cancer cells and A375 melanoma cell xenograft tumor model. About 2×10^6^ tumor cells were implanted subcutaneously on the right hind leg. The tumor volume would arrive to 300~500mm^3^ after 3 weeks later. The tumor-bearing mice were anesthezated by 0.8-1.0μl/g chloral hydrate and injected with 10 μg (100 μl) of the above Dylight755-labeled HPV16 E7-binding affibody molecules, including wild SPA-Z (Zwt) affibody as control, into the tail vein. At least three mice were used in each group. To show that these uptakes were specifically target-mediated of HPV16 E7, HPV18 positive cervical cancer xenograft and HPV negative melanoma xenograft treated with HPV16 E7-binding affibody and HPV16 positive cervical cancer xenograft treated with Zwt affibody were used as control group. At 5 min, 30 min, 1h, 1.5 h, 2 h, 4 h, 6 h, 8 h and 24h after injection, the imaging was performed using the machine of *in vivo* Fluorescence Imaging System (CRi Maesro 2.10). We set the dylight755(NIR) excitation filter (671-705nm) and the second barrier filter (750 longpass), and using the models of 8bit, 2×2, to obtain the fluorescence images from the wavelength of 730nm to 950nm at 10nm interval, data was analyzed by the Maesro software (version 2.10). After finished the total observation of the biodistribution of HPV16 E7-binding affibodies, the tumor-bearing mice were sacrificed, and the main organs as well as tumors were harvested, and their fluorescences were further measured as above. All animal tests and experimental procedures were approved by the Ethical Committee of Wenzhou Medical University and Laboratory Animal Management Committee of Zhejiang Province.

## SUPPLEMENTARY MATERIALS FIGURES


