# Supplementary material for: Generation of affibody molecules specific for HPV16 E7 recognition
Source: Oncotarget. 2016 Sep 21;7(45):73995–4005. doi: 10.18632/oncotarget.12174 (PMC5342030; doi:10.18632/oncotarget.12174)
Supplement: Supplementary file 1 [file oncotarget-07-73995-s001.pdf]

Generation of affibody molecules specific for HPV16 E7 recognition

SUPPLEMENTARY FIGURES

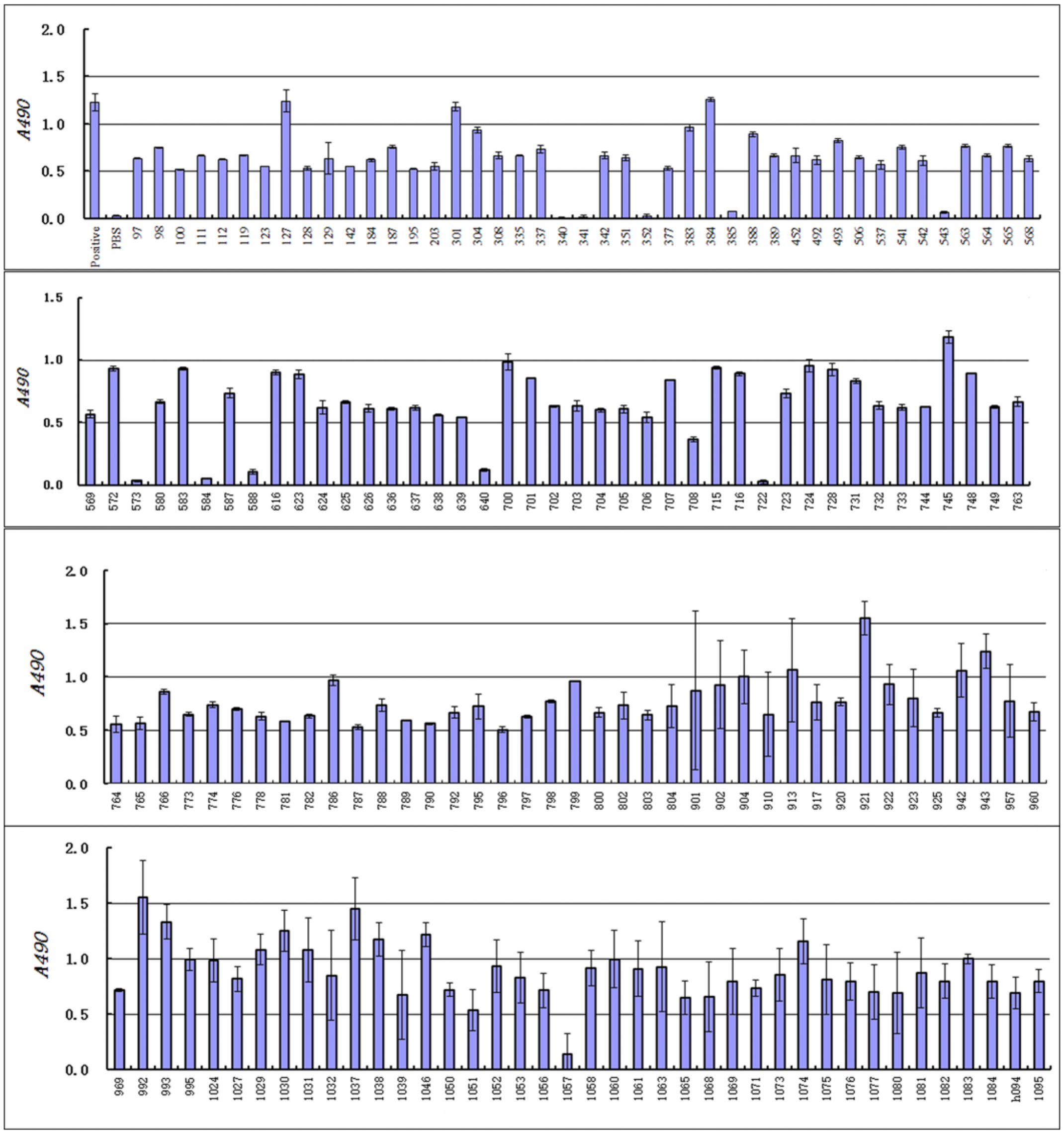

Supplementary Figure S1: ELISA screening for target-binding activity of potential HPV16 E7-binding affibody molecules from 165 clones.

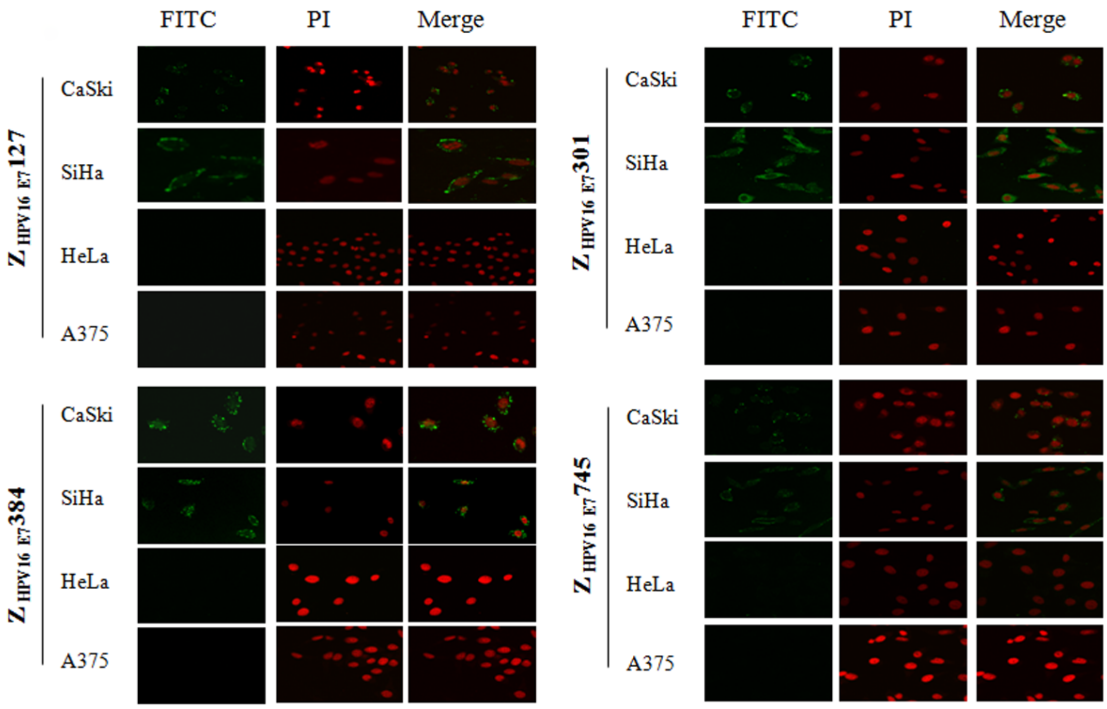

Supplementary Figure S2: Fluorescence staining of HPV16 positive SiHa and CaSki cells with Z $_{HPV16E7}$127, Z $_{HPV16E7}$301, Z $_{HPV16E7}$384 and Z $_{HPV16E7}$745 affibody molecules. Combined with FITC-conjugated goat anti-rabbit IgG, rabbit anti-SPA antibody which recognized wild-type Z domain was used to detect the affibody molecules. The brightly dotted or crumby fluorescence signals were observed in both perinuclear area and nuclear membrane (200×). HPV-negative A375 cell and HPV18 positive HeLa cell were used as controls and did not show any fluorescence signal when the cells were stained with the 4 selected HPV16 E7-binding affibodies. Nuclei were counterstained with PI staining (red).

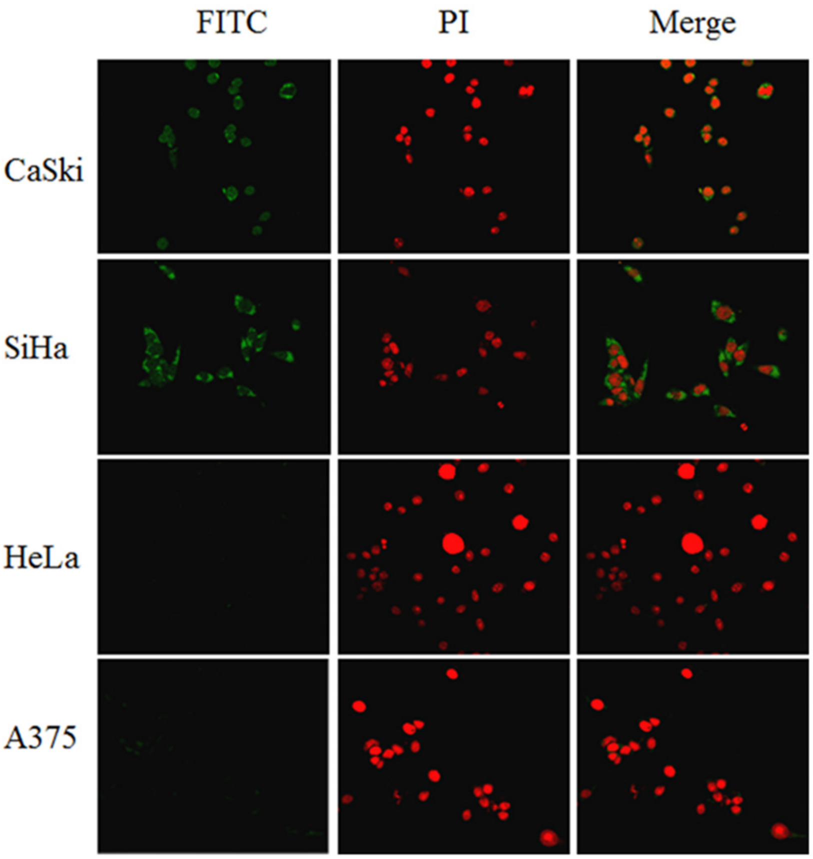

Supplementary Figure S3: Fluorescence staining using anti-HPV16E7 antibody. Fluorescence staining of HPV16 positive SiHa and CaSki cells, HPV18 positive HeLa cell and HPV negative melanoma cell of A375 with anti-HPV16E7 rabbit immune sera, followed with FITC-conjugated goat anti-rabbit secondary antibody. Nuclei were counterstained with PI staining (red).

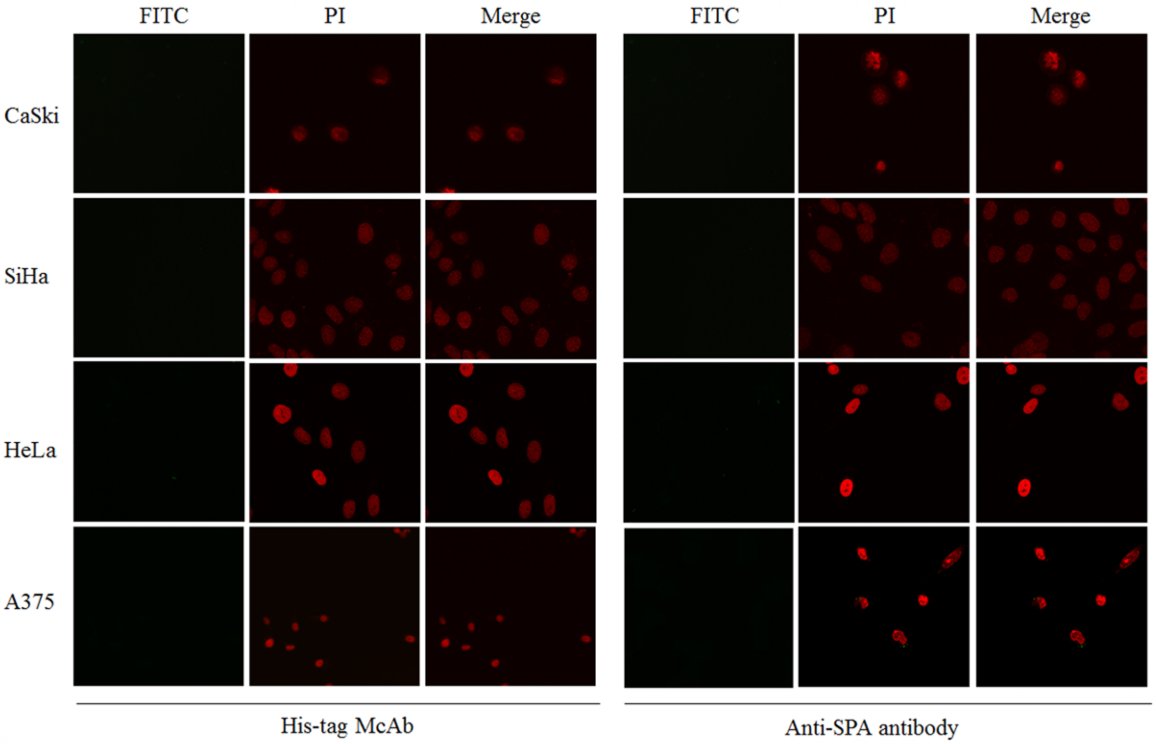

Supplementary Figure S4: Fluorescence staining using wild SPA-Z molecule. Fluorescence staining of HPV16 positive SiHa and CaSki cells, HPV18 positive HeLa cells and HPV negative A375melanoma cells, after incubated with wild SPA-Z molecules for 6h, with anti-his tag mouse monoclonal antibody; followed with FITC-conjugated goat anti-mice secondary antibody. Nuclei were counterstained with PI staining (red).

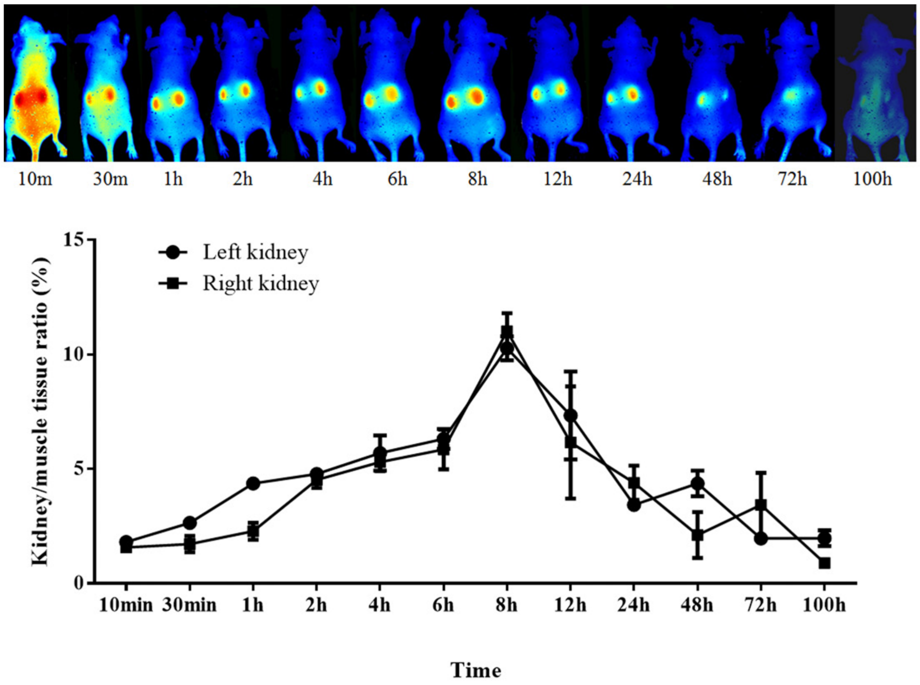

Supplementary Figure S5: *In vivo* biodistribution of Z $_{\text{HPV16E7}}$384 affibody molecule in healthy normal mice. After tail-vein injected with Dylight755- conjugated Z $_{\text{HPV16 E7}}$384 affibody molecule, fluorescence images were obtained from mice at different time points. Kidney uptake was prominent for the accumulation of Z $_{\text{HPV16E7}}$384 affibody molecule. Accumulation of Z $_{\text{HPV16E7}}$384 affibody molecule maximally occurred at 8 h p.i. and then decreased over the time course. The signal was undetectable at 100 h p.i.

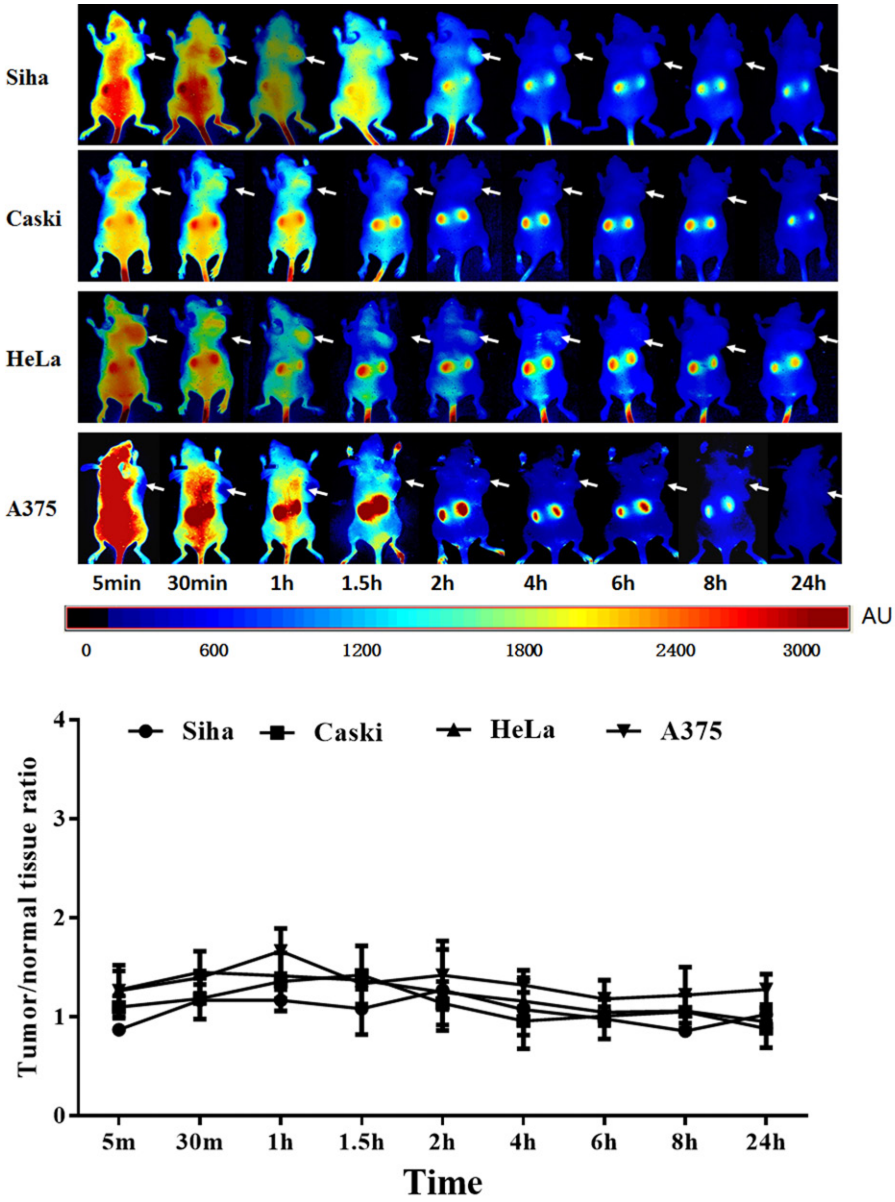

Supplementary Figure S6: Tumor-targeted fluorescence imaging of the wild SPA-Z affibody. A. *In vivo* fluorescence imaging of tumor-bearing mice (arrows) injected with Dylight755- conjugated wild SPA-Z affibody at 5 min, 0.5, 1, 1.5, 2, 4, 6, 8 and 24 h. **B.** Tumor-to-background ratio of mice injected with Dylight755-labeled wild SPA-Z affibody. The data was represented as mean ± standard deviation (SD) of three mice (n = 3) in each group.
